# Agglomerates of nanoparticles

**DOI:** 10.3762/bjnano.11.70

**Published:** 2020-06-02

**Authors:** Dieter Vollath

**Affiliations:** 1 NanoConsulting, Primelweg 3, Stutensee 76297, Germany

**Keywords:** agglomeration, enthalpy, entropy, Gibbs entropy, nanoparticles, size distribution

## Abstract

Nanoparticles tend to agglomerate. The process of agglomeration is ruled by thermodynamics. Depending on the sign of the enthalpy of interaction, ensembles consist of (repelling) poorly agglomerated or (attracting) highly agglomerated particles. For these two cases different distribution functions for the agglomerates were found. The size distribution of the agglomerates is ruled by the maximum of the entropy of the ensemble of agglomerates, which is calculated using Gibbs formula of entropy. The exact determination of the size distribution of the agglomerates also gives the maximum size of the agglomerates. These considerations lead to an improved understanding of ensembles of agglomerated nanoparticles.

## Findings

It is a well-known fact that ensembles of nanoparticles have the tendency to agglomerate. Experimentally working scientists quite often face the problem that it is nearly impossible to find non-agglomerated particles. Typical examples are given in any textbook [1]. To perform electron microscopy of individual particles to analyze their shape or structure, it is necessary to apply a separation process. Earlier, the problem of agglomeration was already discussed in connection with colloids [2–6]. Recently, two studies of the formation of agglomerates of nanoparticles were published [7–8]. In these studies, a distribution of particles exhibiting a maximum of the entropy was sought. These studies resulted in arrangements of particles in which the majority of particles was not found in agglomerates. Furthermore, the results of these studies [7–8] were also applied to experimental results, assuming that not all the particles of an ensemble are equal in size. The free enthalpy *G* of an ensemble with *N* particles showing interaction is given by

[1]
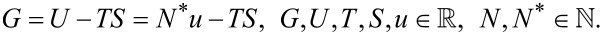


The quantity *U* = N^*^u is the enthalpy of interaction. In an ensemble only N^*^ particles having interaction partners are bound with the energy *u*. This definition is valid in all cases in which the energy of interaction is independent on the number of particles in the agglomerate and not connected to a directed force. Furthermore, it is assumed that most of the agglomerates are large; therefore, it is not necessary to distinguish between particles at the surface and in the center of the agglomerate. The quantity *S* stands for the entropy and *T* is the temperature. The energy *u* of interaction does not depend on the size of the agglomerate and the arrangement of the particles within the agglomerate [9–11].

For the considerations in this paper, details of the local arrangement are not of importance. Therefore, N^*^ = *N* – *N*_1_, where the quantity *N*_1_ is the number of non-interacting particles. The enthalpy of interaction is given by

[2]
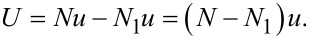


Assuming a maximum size of the agglomerates *N*_max_, the size of the agglomerates follows a discrete distribution function *f*(*i*) with the normalization

[3]
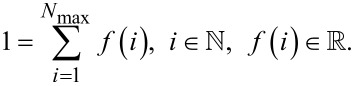


Furthermore, the number of particles in the ensemble is constant; therefore, the sum of the numbers of particles in the agglomerates is constant. This leads to the boundary condition

[4] $$N=\sum_{i=1}^{N_{\max}}\left\lfloor i\;f\left(i\right)\right\rfloor \;.$$

The system of agglomerates is in thermodynamic equilibrium when the free enthalpy *G* at a minimum. A necessary condition for this minimum is a maximum of the entropy given by

[5]
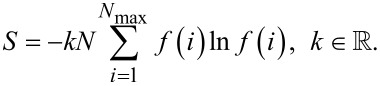


In Equation 5 the quantity *k* stands for the Boltzmann constant. Taking into account the boundary conditions given in Equation 3 and Equation 4, the distribution function can be calculated either pointwise or by assuming an appropriate distribution function. For this study, the Weibull distribution [12],

[6]
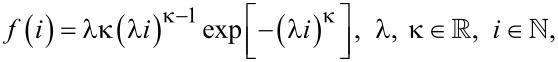


was selected, because by using this distribution law it is possible to approximate nearly all possible courses of distribution functions. In Equation 6, the quantities λ and κ are adjustable parameters for approximation. Taking into account the boundary conditions, the parameters for the Weibull distribution function were determined by an iteration process using nested intervals. The calculations were performed for 10^2^, 10^3^, and 10^4^ particles.

Depending on the starting values, the resulting distribution function showed a maximum at *i* = 1 or *i* = *N*_max_. In both cases, the entropy values were identical. Looking at the equation for the Gibbs entropy (Equation 5) one realizes that the summands are independent on the index *i*. Therefore, one may write

[7]
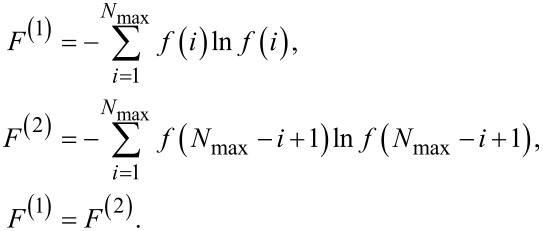


For the following discussions, *F*^(1)^ is attributed to *f*(1) = 1 and *F*^(2)^ to *f*(*N*_max_) = 1. This leads to two distribution functions *P*^(1)^ and *P*^(2)^. These distribution functions are, per definition, related to the free enthalpy values *G*^(1)^ and *G*^(2)^:

[8]
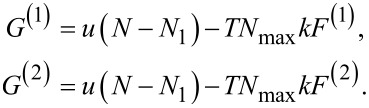


**Figure 1:**
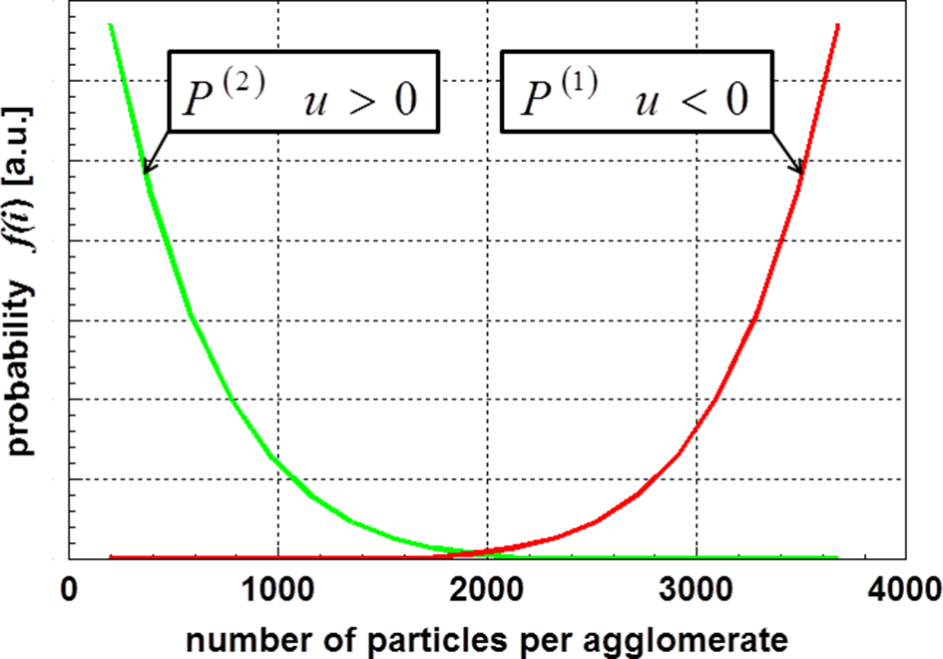
Course of the probability for different sizes of agglomerates. The calculations were performed for 10^4^ particles.

The results of Sokolov et al. [7] and Kätelhön et al. [8] yielded a distribution function of the type *P*^(2)^. Figure 1 shows both distribution functions determined for an ensemble of 10^4^ particles.

Now, one may ask which one of the distribution functions, displayed in Figure 1 correlates with reality. The answer is found using Equation 2. In general, one has to distinguish the following two cases:

[9]
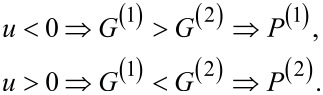


Equation 9 shows that the two different kinds of the probability distribution differ in the sign of the enthalpy of interaction. Table 1 displays the detailed results of the calculation for different sizes of the ensemble of particles. The calculations were performed for three different numbers of particles in the ensemble. The exact number of particles in the ensembles is determined by the fact that for each size of agglomerates this number must be described by an integer. It is important to note that the extrema for the entropy are very flat. The small value of the parameter λ means that the size distribution is very close to a modified exponential distribution.

**Table 1:** Detailed results of the calculations for three different numbers of particles in the ensembles for the distribution function *P*^(1)^. Except for the Weibull parameters, the data are identical for the distribution function *P*^(2)^.

number of particles	size of the largest agglomerate	reduced entropy *S*/*k*	Weibull parameters	

			λ	κ

102	50	314.3	5·10^−8^	5.2
1020	377	5103	5·10^−8^	5.8
10017	3675	70169	3·10^−8^	8.0

**Figure 2:**
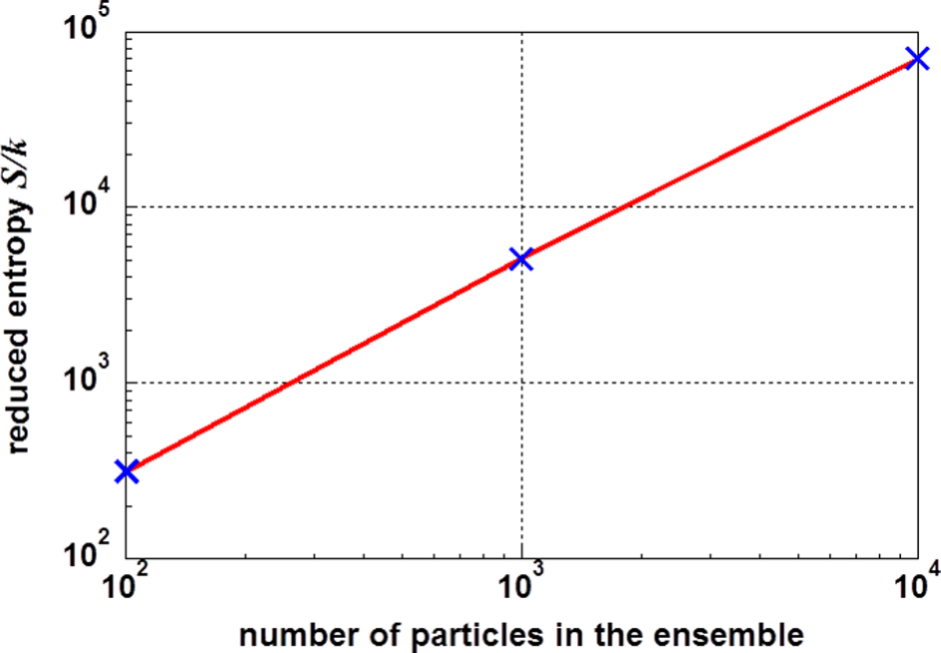
Reduced entropy *S*/*k* as a function of the number of particles in an ensemble. The linear dependency in a double logarithmic system allows for the extrapolation for any number of particles in an ensemble.

**Figure 3:**
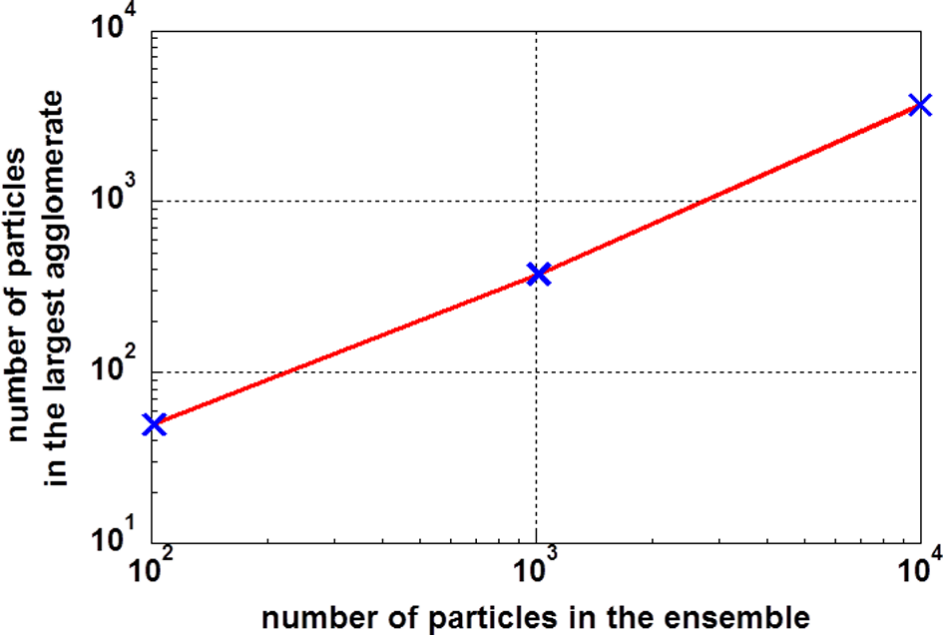
Number of particles in the largest agglomerate as a function of the particle number in the ensemble.

The numbers shown in Table 1 are displayed graphically in the Figure 2 and Figure 3. Figure 2 displays the reduced entropy values *S*/*k* for three different numbers of particles. In a double logarithmic plot, one finds a linear correlation between the logarithm of the number of particles and the logarithm of the entropy. This correlation may be expressed as log(*S*/*k*) = 0.16 + 1.17·log(*N*). This linear correlation allows for the extrapolation for any number of particles in an ensemble.

The second interesting parameter is the number of particles in the largest agglomerate. The size of the largest agglomerate as a function of the number of particles in the ensemble is shown in Figure 3. It is interesting to see that this function is also linear in a double logarithmic plot. It can be expressed by the formula log(*N*_max_) = −0.22 + 0.94·log(*N*). When the distribution function *P*^(2)^ applies, there is at least one agglomerate of the maximum size.

One may summarize the results of the presented study as follows: The enthalpy of interaction between the particles determines the size distribution of the agglomerates. Depending on the sign of the enthalpy of interaction, the maximum of the distribution function is either at the state of non-agglomerated particles or at the entropy and size of the largest particle agglomerates. The calculations show that, depending on the number of particles within the ensemble, the size of the largest agglomerate within an ensemble follows a linear relation in a double logarithmic system.
